# Beyond ATP: Lipid-Driven Plasticity and the Immunometabolism of ILC2s

**DOI:** 10.3390/cells15090838

**Published:** 2026-05-03

**Authors:** Vanessa-Vivien Pesold, Jafar Cain, Steven J. Bensinger, Omid Akbari

**Affiliations:** Department of Immunology and Immune Therapeutics, University of Southern California, Los Angeles, CA 90033, USA; jc_769@usc.edu (J.C.);

**Keywords:** ILC2, lipid metabolism, fatty acid oxidation, immunometabolism, IL-33

## Abstract

Group 2 innate lymphoid cells (ILC2s) are tissue-resident immune cells that play a central role in type 2 immunity. Beyond cytokine signaling, they integrate inputs from lipids, nutrients, neuroendocrine mediators, and local metabolic cues, establishing cellular metabolism as a key regulator of their function. Immunometabolism provides a framework to understand how ILC2s adapt to diverse tissue environments such as the lung, adipose tissue, gut, skin, and brain, each defined by distinct nutrient availability, oxygen tension, and inflammatory conditions. Unlike many immune cells that primarily rely on glycolysis, ILC2s dynamically balance glycolysis, fatty acid oxidation (FAO), and oxidative phosphorylation (OXPHOS) depending on activation state and tissue context. Lipids not only serve as energy substrates but also regulate membrane organization, lipid raft–dependent signaling, and the generation of bioactive mediators, including eicosanoids, oxysterols, and sphingolipids. Emerging evidence linking cholesterol biosynthesis, steroid metabolism, and sphingolipid signaling to ILC2 function underscores the importance of lipid-dependent immune regulation. Dysregulation of these pathways contributes to chronic inflammatory diseases such as asthma, metabolic disorders, and fibrosis. Targeting metabolic pathways and checkpoints may therefore offer new strategies to modulate ILC2-driven pathology. This review summarizes current insights into metabolic programs governing ILC2 activation, survival, and plasticity and highlights emerging therapeutic opportunities.

## 1. Introduction

Innate lymphoid cells (ILCs) are a heterogeneous population of early-responding immune cells within the innate immune system. Based on their developmental origin, transcription factors, surface receptors, and cytokine profiles, ILCs are classified into three main groups: ILC1s, ILC2s, and ILC3s. Distinct ILC subsets exhibit disease-specific functional phenotypes. ILC1s are linked to type 1 inflammation and anti-tumor immunity, ILC2s to allergic disease, tissue repair, and metabolic homeostasis, whereas ILC3s contribute to barrier defense and chronic inflammatory disorders. These roles are further shaped by subset-specific metabolic programs and tissue microenvironments [1,2]. After their generation in the bone marrow and maturation in the thymus, ILCs populate peripheral tissues, where they function predominantly as tissue-resident cells maintaining barrier integrity and responding to local inflammatory stimuli. Their egress into the circulation is largely mediated by sphingosine-1-phosphate (S1P) receptor signaling [1,2,3]. Among these subsets, ILC2s are most strongly associated with disease. They resemble T helper (Th) 2 cells but lack antigen-specific receptors and classical leukocyte lineage markers [1,4,5]. Despite this, ILC2s can exhibit memory-like properties and respond more rapidly upon repeated stimulation [6,7]. They act as a critical link between innate and adaptive immunity and are activated by a broad spectrum of inflammatory, neuroendocrine, and non-cytokine signals, including lipids, peptides, and hormones [1,8,9]. ILC2s are enriched in barrier tissues such as the lung, skin, gastrointestinal tract, and adipose tissue, where they contribute to type 2 immunity, allergic inflammation, host defense against helminths, and tissue repair [1,2]. Dysregulated ILC2 responses drive diseases through the production of cytokines such as IL-5, IL-13, IL-9, and amphiregulin [1,4,5]. ILC2s function within broader innate immune networks that critically shape disease development. Epithelial alarmins such as interleukin-33 (IL-33), IL-25, and thymic stromal lymphopoietin (TSLP) rapidly activate ILC2s following tissue injury or allergen exposure, while lipid mediators derived from mast cells and myeloid cells, including prostaglandin (PG) D_2_ and cysteinyl leukotrienes, further amplify their responses. In turn, ILC2-derived IL-5 and IL-13 promote eosinophilia, mucus production, airway hyperresponsiveness, alternative macrophage activation, and tissue remodeling, thereby linking early innate sensing to chronic inflammatory pathology [2,9]. To support these functions, ILC2s generate adenosine triphosphate (ATP) through both mitochondrial pathways, such as the tricarboxylic acid (TCA) cycle and FAO, and non-mitochondrial glycolysis. These pathways are tightly interconnected and regulated by signaling networks including the mechanistic target of rapamycin (mTOR) pathway [10,11]. However, tissue-specific microenvironments differ substantially in nutrient availability, oxygen tension, and pH, requiring ILC2s to maintain a high degree of metabolic flexibility [10,11,12]. Unlike many immune cells that predominantly rely on glycolysis, ILC2s frequently depend on lipid metabolism for energy generation [10,11,13]. While glycolytic activity is relatively low at baseline, it is strongly upregulated upon activation in a manner resembling the “Warburg effect,” supporting cytokine production, proliferation, and cellular interactions [10,11]. Notably, FAO is concurrently enhanced, highlighting a coordinated metabolic program rather than a simple metabolic switch. This so-called “dichotomous metabolism” enables ILC2s to adapt efficiently to changing environmental and functional demands [11,13,14]. Beyond ATP production, metabolism fundamentally shapes immune function. This concept, referred to as immunometabolism, has become a central focus in immunology, with interconnected pathways regulated by immunometabolic checkpoints [10].

This review summarizes current insights into ILC2 immunometabolism under homeostatic and pathological conditions, with particular emphasis on the interplay between immune function and lipid metabolism. Emerging evidence highlights that lipids act not only as structural components but also as key signaling molecules and bioactive mediators in immune regulation [10,11,13,15].

## 2. Central Metabolic Pathways in ILC2s

Cellular energy metabolism comprises interconnected pathways that generate ATP and biosynthetic intermediates required for cellular function.

Glycolysis converts glucose to pyruvate and supports rapid ATP generation, whereas mitochondrial pathways, including the TCA cycle and OXPHOS, provide sustained energy production. The pentose phosphate pathway (PPP) supplies nicotinamide adenine dinucleotide phosphate (NADPH) for redox balance and ribose-5-phosphate for nucleotide synthesis [16,17,18,19,20,21,22,23]. In parallel FAO degrades fatty acids (FAs) in mitochondria to generate acetyl-coenzyme A (acetyl-CoA), nicotinamide adenine dinucleotide (NADH), and flavin adenine dinucleotide (FADH_2_), thereby fueling the TCA cycle and OXPHOS. Conversely, FA synthesis and exogenous lipid uptake support membrane biosynthesis, signaling, and intracellular energy storage [23,24,25,26,27,28]. Glutamine can further replenish TCA cycle intermediates through anaplerosis [29]. Although these pathways are conserved across immune cells, their relative utilization is context-dependent. ILC2 metabolism is highly flexible and not regulated cell-intrinsically alone but is continuously shaped by the surrounding tissue microenvironment. Stromal-cell-derived IL-33, local lipid availability, oxygen tension, neuronal mediators, and microbiota-derived metabolites can directly influence ILC2 activation state, substrate preference, and effector function. In adipose tissue, reciprocal crosstalk between stromal cells and ILC2s supports metabolic homeostasis, whereas obesity-associated inflammation disrupts these circuits and promotes ILC2 dysfunction [1,2,16,18,28]. While glycolysis increases upon activation, ILC2s rely strongly on lipid metabolism to sustain effector function and cellular plasticity. FAO provides reducing equivalents for OXPHOS and supports mitochondrial fitness [30,31]. This distinguishes ILC2s from ILC1 and ILC3 subsets, which more strongly depend on glycolytic metabolism [31,32]. A central regulator of this lipid-driven program is peroxisome proliferator-activated receptor γ (PPARγ), which promotes lipid uptake, FA utilization, and optimal responsiveness to IL-33. PPARγ is highly expressed in lung and adipose tissue ILC2s and enhances IL-5 and IL-13 production [30,33]. Mechanistically, CD36 and FA transport proteins further support intracellular lipid utilization in activated ILC2s [28,30,32,33,34,35,36] (Table 1).

## 3. The Lipid Landscape: Energy, Structure, Storage and Signaling

### 3.1. Quiescent Versus Activated ILC2s

In the quiescent state, ILC2s express low levels of activation markers, produce minimal amounts of cytokines, and exhibit limited proliferation. However, these cells are not metabolically inactive; instead, they maintain a low-demand, survival-oriented program that supports long-term tissue residency, mitochondrial fitness, and rapid responsiveness to future stimulation. To sustain homeostasis under nutrient-restricted conditions while minimizing reactive oxygen species (ROS), they predominantly rely on FAO. Through β-oxidation, FAO generates acetyl-CoA and reducing equivalents that fuel the TCA cycle and OXPHOS, thereby supporting long-term survival without excessive lactate accumulation [23,24]. FAs are taken up via transporters such as CD36 and fatty acid transport proteins (FATPs), transported into mitochondria via the carnitine shuttle carnitine palmitoyltransferase 1 (CPT1), and metabolized through β-oxidation, the TCA cycle, and OXPHOS. This program is supported by amino acid uptake, including valine, isoleucine, glutamine, and arginine, while glycolysis remains low [13,14,23,47,48,49,50,51]. PPARγ serves as a central regulator of lipid metabolism by promoting FAO and lipid uptake, while Adenosine monophosphate-activated protein kinase (AMPK) supports catabolic metabolism and mTOR activity remains low, thereby limiting proliferation [30,33,49,50,51,52]. This metabolic profile resembles that of memory T cells and is optimized for survival and tissue residency [11,53,54]. Although less well characterized, ILC1s and ILC3s in resting states also primarily utilize FAO and OXPHOS, in contrast to many effector T helper cells that rely on glycolysis [11,31,48,53,55].

Upon activation by alarmins ILC2s rapidly increase cytokine production and biomass generation [1,5,56]. This transition is accompanied by increased glycolytic flux, enabling rapid ATP generation and provision of biosynthetic intermediates required for proliferation and effector cytokine production. Inflammatory glycolysis may further stabilize hypoxia-inducible factor 1 alpha (HIF-1α) and amplify ILC2 responses under hypoxic airway conditions [18,20,36]. Despite this increased demand, they continue to rely heavily on FAO, which supports ATP production, energy homeostasis, and survival [11,15,23,32,56]. FA uptake is further enhanced under conditions such as helminth infection or vitamin A deficiency, and activated ILC2s display higher FA uptake than ILC3s [13,22,57,58]. In parallel, amino acid metabolism supports mitochondrial function and effector responses. Activated ILC2s exhibit increased intracellular levels of amino acids, including alanine, valine, leucine, and isoleucine, which are imported via transporters such as SLC7A5, SLC7A8, SLC3A2, and SLC43A2. It was shown that deficiency of SLC7A8 impairs ILC2 function by reducing OXPHOS and mTOR signaling [14,47,59]. Arginine metabolism via arginase-1 (ARG1) further promotes proliferation, collagen synthesis, and airway hyperresponsiveness [59]. In addition, a mitochondrial signal transducer and activator of transcription (STAT) 3–methionine metabolic axis supports ATP production and effector function following IL-33 stimulation [60]. Notably, glycolysis is also upregulated upon activation, accompanied by increased expression of glucose transporter 1 (GLUT1) and glycolytic enzymes [14,36]. However, rather than switching exclusively to glycolysis, ILC2s co-regulate glycolysis and OXPHOS. This “dichotomous metabolism” enables flexible adaptation to environmental and functional demands, with OXPHOS supporting proliferation and survival, and glycolysis primarily fueling cytokine production [14]. Hence, ILC2s display a coordinated use of glycolysis and mitochondrial oxidative pathways, including FAO and OXPHOS, rather than a strict binary switch between exclusive states. In these cells, the balance between these programs changes according to activation state, tissue niche, and nutrient availability [14,48]. Adequate mitochondrial function further prevents apoptosis and supports cellular longevity [17,61]. Beyond energy production, metabolic reprogramming supports structural and biosynthetic demands during activation. Early phases involve increased FA uptake and membrane remodeling, including lipid raft reorganization and receptor clustering. This is followed by transient lipid storage in lipid droplets (LDs) and subsequent upregulation of anabolic lipid pathways, culminating in phospholipid synthesis and membrane expansion [13,34,35,62]. These processes are initiated by IL-33 in a glucose-dependent manner and regulated by mTOR and PPARγ [30,49,52]. ILC2 metabolism is further shaped by nutrient availability and environmental cues. In addition to FAs and glucose, branched-chain amino acids (BCAAs), aryl hydrocarbon receptor (AhR) ligands, vitamins, and minerals contribute to ILC2 function, while factors such as diet, microbiota, and circadian rhythms further modulate metabolic programs [21,53,63,64]. Importantly, ILC2 metabolic profiles vary across tissues. Lung ILC2s express high levels of the IL-33 receptor ST2 and are primed for rapid activation, whereas skin ILC2s reveal lower ST2 expression [1,2,65]. Adipose tissue ILC2s interact with adipocytes, recruit eosinophils, and contribute to metabolic homeostasis [66]. However, how metabolic programs differ across tissues and stimuli remains an active area of investigation [1,5,31]. Studies by Akbari et al. indicate that baseline expression profiles, activation states, and functional roles of ILC2s vary substantially depending on the tissue microenvironment [67].

**Figure 1:**
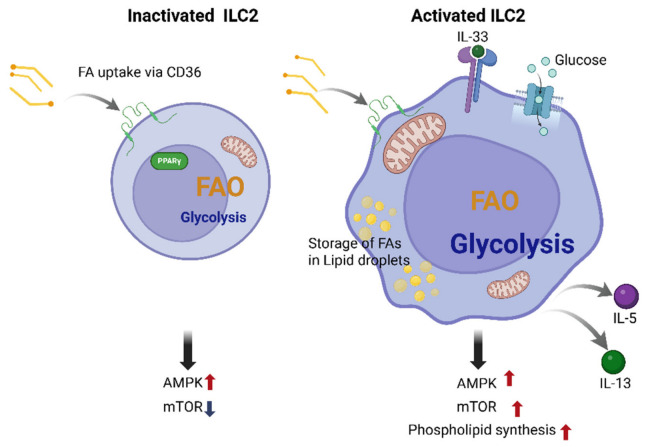
Metabolic reprogramming of ILC2s. Inactivated ILC2s primarily rely on FAO to maintain mitochondrial respiration, regulated by CD36-mediated lipid uptake, PPARγ, and AMPK. Upon IL-33 stimulation, ILC2s adopt a “dichotomous metabolism” characterized by concurrent FAO and glycolysis, supporting lipid remodeling, biomass expansion, and cytokine production. Created with BioRender.com (2026). Accessed on 10 April 2026.

Notably, apparent discrepancies regarding the relative dependence of ILC2s on glycolysis versus FAO likely reflect context-dependent metabolic programming rather than true contradictions. ILC2 metabolism is shaped by tissue localization, nutrient availability, inflammatory duration, and activating signals [47,48,53,55,56]. For example, lung-resident ILC2s may transiently increase glycolysis to support rapid cytokine production and proliferation, whereas adipose-resident ILC2s in lipid-rich environments more strongly rely on FAO-driven oxidative metabolism [49,52,66,67]. Likewise, intestinal ILC2s during helminth infection or nutrient stress can increase exogenous FA uptake and oxidative metabolism to preserve barrier immunity [58]. Thus, glycolysis and FAO should not be viewed as mutually exclusive pathways, but as flexible and coordinated modules whose relative contribution varies according to microenvironmental and functional demands [47,48,53] (Figure 1).

### 3.2. Storage of Lipids in Activated ILC2s

Accumulation of free FAs in adipose tissue ILC2s can induce lipotoxicity and metabolic stress, which can be compensated by increased glucose uptake [68]. For further prevention of lipotoxicity, FAs are esterified into TAGs and stored in LDs [37,68]. These lipid stores function as dynamic reservoirs that buffer FA availability, support membrane biosynthesis, and provide substrates for sustained mitochondrial metabolism during inflammation [34,37]. LDs consist of a hydrophobic core of TAGs and cholesterol esters surrounded by a phospholipid monolayer with associated proteins [37,69]. Under nutrient deprivation, LDs redistribute along microtubules and form contact sites with mitochondria, enabling efficient FA supply for FAO [70]. LD morphology varies with activation state, appearing spherical after IL-2 stimulation and more irregular following IL-33 activation. In addition to serving as energy reservoirs, lipids provide essential building blocks for membrane synthesis [34,37,69]. Transient storage in small LDs allows rapid mobilization via lipolysis or lipophagy, whereas larger LDs in highly active ILC2s likely reflect increased lipid turnover [13,69]. LD formation is regulated by cytokines and metabolic signals. IL-33 promotes FA uptake and lipid accumulation and may modulate transporter expression (e.g., CD36) and mitochondrial activity [17,19]. Lipid-sensitive regulators such as PPARγ and diacylglycerol O-acyltransferase 1 (DGAT1) coordinate lipid uptake, synthesis, and FAO, while linking these processes to glycolysis and mTOR signaling [13,31]. During airway inflammation, uptake of exogenous lipids is further enhanced, amplifying ILC2 activation. This process is driven by IL-33 and mTOR signaling, which upregulate PPARγ and DGAT1 in a glucose-dependent manner. Uptake of exogenous FAs appears more efficient for ILC2 proliferation than de novo synthesis, while LD formation prevents lipotoxicity during high lipid influx [34]. Overall, FA uptake, storage, and utilization are tightly coordinated. Nutrient availability determines whether FAs are stored, oxidized, or used for biosynthesis. Under high glucose conditions, FAs preferentially support membrane remodeling, whereas under low glucose or increased energy demand, they are mobilized for β-oxidation. In lipid-rich or inflamed environments, ILC2s increase lipid storage to avoid lipotoxicity while maintaining FAO capacity [31,70,71].

## 4. Membrane Architecture: Lipids as Regulators of Signaling Sensitivity

### 4.1. Cholesterol Metabolism in ILCs and Other Immune Cells

Cholesterol metabolism was long considered a structural process of membrane maintenance but is now recognized as a key regulator of immune cell function. Cellular cholesterol levels are tightly controlled through de novo biosynthesis, uptake, storage, and efflux, largely governed by sterol regulatory element-binding proteins (SREBPs). De novo synthesis originates from acetyl-CoA via the mevalonate pathway and is regulated by SREBP2, while extracellular cholesterol is acquired through lipoprotein uptake, enabling dynamic adaptation to metabolic demand [26,72]. A recent study has identified a direct link between cholesterol metabolism and ILC2 function. In mice and human ILC2s, the costimulatory molecule inducible T-cell costimulator (ICOS) was shown to restrain cholesterol biosynthesis and IL-10 production. Loss of ICOS enhanced SREBP2-driven cholesterol synthesis and increased IL-10 production, indicating that cholesterol availability shapes ILC2 functional polarization. Consistently, inhibition of cholesterol synthesis or transport reduced IL-10 levels, whereas cholesterol availability supported cortisol biosynthesis. Upregulation of steroidogenic enzymes (e.g., CYP11A1) and the glucocorticoid receptor (GR) linked cholesterol metabolism to transcription factors such as MAF bZIP transcription factor (c-MAF) and nuclear factor interleukin-3-regulated (NFIL3), which regulate IL-10 expression [9,14]. In addition, small amounts of cortisol secretion by ILC2s and a potential role of ICOS in steroid resistance have been suggested [9,15]. These findings support a model in which cholesterol metabolism promotes an IL-10–producing regulatory ILC2 phenotype. Evidence from other immune cell types further underscores the role of cholesterol metabolism in immune regulation. In T cells, inhibition of cholesterol biosynthesis reduced IL-10 expression through decreased c-MAF activity [26,72]. In tumor-infiltrating T cells, cholesterol deficiency impaired proliferation and induced apoptosis, while oxysterols promoted dysfunction via liver X receptor (LXR) and SREBP2 signaling [73]. Conversely, cholesterol accumulation in aged T cells supported inflammation and reduced apoptosis, contributing to atherosclerosis in mice [74]. In natural killer (NK) cells, myocyte enhancer factor 2C (MEF2C) regulates lipid metabolism via SREBP signaling, influencing cellular function [75]. Elevated cholesterol levels and high-fat diets are broadly associated with immune dysregulation and inflammatory disease [26,72]. Trindade et al. reported that cholesterol metabolites such as 25-hydroxycholesterol can suppress differentiation of immunoglobulin A (IgA)-producing plasma cells through SREBP2 regulation [39].

Overall, these findings establish cholesterol metabolism as a central regulator of immune function. In ILC2s, cholesterol-dependent pathways contribute to the induction of a regulatory IL-10–producing phenotype. Consistent with this, IL-10 production is linked to metabolic reprogramming, as a shift from FAO to glycolysis enhances IL-10 expression via IL-4 receptor (IL-4Rα) signaling and STAT6-dependent upregulation of c-MAF and B-lymphocyte-induced maturation protein 1 (Blimp-1), thereby limiting IL-5 and IL-13 production and restraining ILC2 effector function [76].

### 4.2. Lipid Rafts

**Figure 2:**
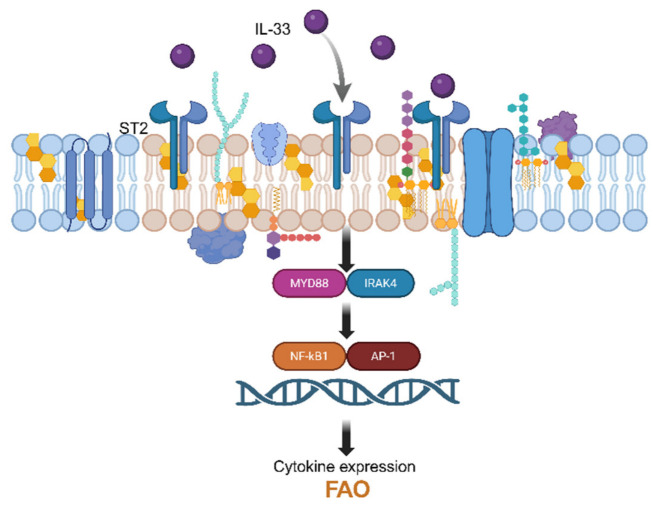
Lipid raft–dependent regulation of IL-33 signaling in ILC2s. Cholesterol-rich lipid rafts promote clustering of the IL-33 receptor ST2 and activate NF-κB and AP-1. Membrane lipid organization thereby controls signaling strength and ILC2 effector function. Created with BioRender.com (2026). Accessed on 10 April 2026.

The plasma membrane contains specialized domains that enable lateral organization, known as lipid rafts. These structures consist of coexisting liquid phases: a liquid-disordered phase (Ld), enriched in unsaturated phospholipids, and a liquid-ordered phase (Lo), enriched in saturated phospholipids, sphingolipids, and cholesterol. Lipid rafts are small, dynamic membrane domains (~10–200 nm) that arise from lipid phase separation driven by differences in acyl chain length and saturation, as well as lipid–lipid and lipid–protein interactions [35,77,78]. In addition to phospholipids and cholesterol, lipid rafts contain sphingolipids, lipid-anchored proteins (e.g., glycosylphosphatidylinositol (GPI)-anchored proteins), and specific transmembrane proteins [35,79]. Their interaction with the cytoskeleton supports coordinated processes such as migration and adhesion, while immune receptors integrate raft organization into inflammatory signaling [79,80]. Structurally, lipid rafts include flat, flotillin-rich domains and invaginated, caveolin-rich caveolae (50–100 nm), which participate in endocytic transport [79,81,82]. By selectively enriching or excluding signaling molecules, lipid rafts function as platforms for signal transduction. Key components such as Src family kinases, CD28, Lck, and programmed death-ligand 1 (PD-L1) are frequently localized within these domains [80,83,84]. Rather than acting as simple on/off switches, lipid rafts modulate signaling strength, functioning as a “volume control” of receptor signaling. This principle is particularly relevant in ILC2s, where the IL-33 receptor ST2 clusters within lipid rafts together with its coreceptor interleukin-1 receptor accessory protein (IL-1RAcP), enhancing sensitivity to IL-33 and promoting downstream nuclear factor kappa-light-chain-enhancer of activated B cells (NF-κB) and MAP-kinase (MAPK) signaling [79,80]. Thus, membrane lipid composition directly regulates cytokine responsiveness without altering receptor expression [35,78,79]. In tissues with fluctuating lipid availability, such as lung, gut, or adipose tissue, this mechanism may determine whether IL-33 induces tissue repair or pathological type 2 inflammation. Lipid rafts therefore represent central immunometabolic checkpoints of ILC2 activation [79,80]. Consistent with this, IL-33 has been suggested to promote recruitment of ST2 into lipid rafts in epithelial cells [71,85] (Figure 2).

## 5. Bioactive Lipid Mediators: Control of Activation, Survival, and Inflammation

Lipids fulfill multiple interconnected roles in ILC2 biology, acting as energy substrates, structural components, and bioactive signaling molecules that integrate environmental cues with metabolic state to regulate activation and effector responses. Beyond their structural role, membrane lipids function as signaling molecules, for example via the PI3K–PIP_3_–AKT pathway. Phosphoinositide 3-kinases (PI3Ks) convert phosphatidylinositol 4,5-bisphosphate (PIP_2_) to phosphatidylinositol 3,4,5-trisphosphate (PIP_3_), enabling recruitment and activation of Protein kinase B (AKT) through phosphoinositide-dependent kinase-1 (PDK1) and mTOR complex (mTORC) 2. Activated AKT regulates metabolic pathways controlling glucose uptake, lipid synthesis, and mitochondrial activity. In immune cells, this pathway is closely linked to mTOR signaling and integrates metabolic and activation cues. In ILC2s, signals downstream of receptors such as ST2 and ICOS converge on the PI3K–AKT–mTOR axis, promoting proliferation, survival, and cytokine production [52]. In addition, lipid modifications such as N-myristoylation, S-palmitoylation, and prenylation facilitate membrane association of signaling proteins and assembly of receptor-proximal signaling complexes [62]. Membrane lipids can also be converted into secondary messengers such as arachidonic acid via phospholipase and cyclooxygenase (COX) pathways, generating bioactive mediators including eicosanoids and phosphoinositides [9,86]. These lipid-derived mediators act as integrators of environmental and metabolic signals, linking nutrient availability to intracellular signaling pathways that regulate immune cell activation and differentiation [38,87].

Prostaglandins (PGs) and related lipid mediators exert context-dependent effects on ILC2 function. Activating signals include PGD_2_, cysteinyl leukotrienes (LTC_4_, LTD_4_, LTE_4_), LTB_4_, and neuromedin U (NMU), which are produced during allergic responses and promote ILC2 activation via Ca^2+^/calcineurin/nuclear factor of activated T cells (NFAT) signaling [9,41,42]. This is coupled to metabolic reprogramming, as NFAT-dependent transcription enhances glycolysis and mitochondrial function [38,88]. In contrast, regulatory mediators such as PGE_2_, PGI_2_, maresin-1, and lipoxin A_4_ suppress ILC2 activity through cyclic adenosine monophosphate (cAMP)/protein kinase A (PKA) signaling, likely leading to inhibition of STAT5, cellular myelocytomatosis oncogene (c-Myc), and anabolic metabolism [9,86]. These opposing signals establish a functional continuum between activation and suppression, in which lipid mediators coordinate metabolic pathways with transcriptional programs controlling immune cell fate [38,87,89]. In ILC2s, this balance determines whether cells adopt a pro-inflammatory effector phenotype or a metabolically restrained regulatory state. More broadly, lipid metabolism emerges as a central regulator of immune cell differentiation, actively shaping lineage commitment, survival, and effector function in response to environmental cues [87,89].

Short-chain fatty acids (SCFAs), including acetate, propionate, and butyrate, are produced by gut microbiota and act as important regulators of ILC2 function. SCFAs, particularly butyrate, suppress ILC2 proliferation and airway hyperresponsiveness [63,64]. Mechanistically, SCFAs inhibit histone deacetylases (HDACs), thereby altering chromatin accessibility and suppressing GATA3 activity and cytokine production [90]. In addition, SCFAs act via G protein–coupled receptors to reduce antigen presentation and dampen Th2 responses [63,64]. SCFAs also directly reprogram ILC2 metabolism by activating AMPK, uncoupling protein 2 (UCP 2), and receptors such as free fatty acid receptors (FFAR)2 and FFAR3, promoting mitochondrial respiration and shifting lipid metabolism toward utilization rather than storage [11,64,91,92]. Notably, SCFA effects are tissue- and subset-specific: while butyrate suppresses airway ILC2s, it can increase ILC3 and ILC1 populations in the gut [33,64]. Overall, lipid mediators act as central integrators of environmental signals and intracellular metabolic programs. Activating mediators promote NFAT-driven transcription and metabolic activation, whereas suppressive mediators limit STAT5 signaling and bioenergetic activity. Through this balance, lipid mediators regulate not only ILC2 activation but also the magnitude and metabolic cost of type 2 immune responses [9,41,42,86].

## 6. Sphingolipids

**Figure 3:**
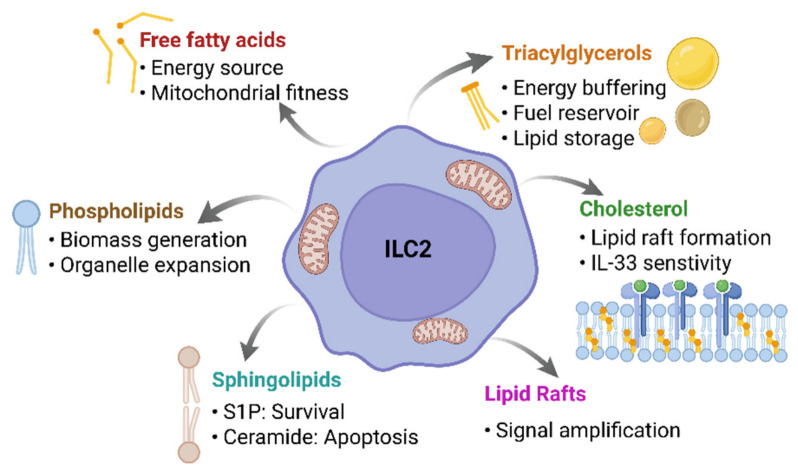
Lipid classes and their functional roles in ILC2s. Distinct lipid species regulate ILC2 metabolism and function beyond energy provision. Free fatty acids fuel mitochondrial respiration, while triacylglycerols provide transient energy storage. Phospholipids support membrane expansion, cholesterol organizes lipid rafts to modulate receptor signaling, and sphingolipids act as bioactive regulators controlling cell fate decisions. Created with BioRender.com (2026). Accessed on 10 April 2026.

Sphingolipid metabolism is closely linked to glycolysis and FAO, acting as a regulatory interface between energy availability, cell fate, and immune function [44,45]. Unlike other lipids, sphingolipids are not used as energy substrates but function as structural membrane components and bioactive signaling molecules [44,93]. This is particularly relevant for immune cells such as ILC2s, which must rapidly adapt to changing metabolic and inflammatory environments without relying primarily on a complete glycolytic switch [2]. De novo synthesis occurs in the endoplasmic reticulum (ER), where ceramides are generated and further processed into sphingomyelin, glycosphingolipids, ceramide-1-phosphate, or S1P, with additional recycling via salvage pathways [44,45,94]. Ceramides and S1P form a functional antagonistic system known as the “sphingolipid rheostat,” in which ceramides promote stress responses and apoptosis, whereas S1P supports survival, proliferation, and migration via PI3K/AKT and MAPK signaling [94,95,96,97]. High ceramide levels act as metabolic “brakes,” dampening insulin signaling, glucose uptake, OXPHOS, and FAO, thereby limiting cellular stress and preventing exhaustion [96,98,99]. In ILC2s, this likely contributes to maintenance of an FAO- and OXPHOS-dominated metabolic state associated with enhanced stress resistance and long-term survival [2,99]. Sphingolipids also regulate immune-cell positioning and signaling. Ceramides promote inflammatory pathways such as c-Jun N-terminal kinase (JNK) and NF-κB, whereas S1P controls survival, chemotaxis, and tissue distribution through S1P receptors (S1PR1–5) [97,98,100,101]. The S1P gradient between blood, lymph, and tissues drives immune-cell trafficking, including ILC2 egress and homing via S1PR1 [97,102]. Receptor-specific effects further refine positioning, as S1PR1 promotes migration, whereas S1PR2 counteracts this by inhibiting Rac and AKT signaling [101]. Dysregulation of sphingolipid metabolism contributes to allergic disease and asthma. Elevated sphingolipid levels are observed in patients, whereas S1P deficiency reduces disease severity [97,103]. Several studies have demonstrated that S1P promotes airway remodeling, bronchial hyperresponsiveness, mast-cell activation, and eosinophil infiltration through calcium-dependent signaling [97,103,104,105]. Pharmacological targeting of this pathway, for example with Fingolimod (FTY720), reduces ceramide levels, Th2 activation, airway hyperresponsiveness, and remodeling [2,106]. Similarly, sphingosine analogs and ceramide-based approaches modulate immune responses and epithelial cell behavior [107,108]. In this context, Sudhadevi et al. hypothesized that S1P does not directly cause bronchoconstriction but rather enhances airway smooth-muscle responsiveness to agonists such as methacholine and histamine [103]. These findings highlight sphingolipid metabolism as a key driver of inflammatory and remodeling processes in the lung (Figure 3).

## 7. The Tissue “Fuel Map”: In Situ Adaptation

In adipose tissue, ILC2s play essential roles in physiological regulation and protect against metabolic diseases such as obesity and diabetes. These cells reside in close proximity to adipocytes within a lipid-rich and relatively hypoxic environment and are therefore metabolically adapted to utilize local lipids via FAO, largely under the control of PPARγ signaling [2]. In white adipose tissue (WAT), ILC2 activation by IL-33 promotes UCP1 expression and induces “beiging,” thereby increasing energy expenditure. This process is further supported by IL-5 and IL-13 production, eosinophils, and IL-4-Receptor-α signaling in adipocyte progenitors [55,66]. Loss of IL-33 signaling impairs ILC2 abundance, WAT beiging, and glucose homeostasis, and promotes a shift toward an ILC1-like phenotype associated with inflammation and metabolic dysfunction. Increased CD36-mediated FA uptake in this context contributes to lipid accumulation and recruitment of pro-inflammatory immune cells [109]. Painter et al. demonstrated that metabolic disorders such as obesity and type 2 diabetes disrupt ILC2 homeostasis, creating a detrimental feedback loop in which elevated free FAs and hyperglycemia induce metabolic stress and reprogram ILC2 metabolism [67]. In murine studies, the obesity-associated adipokine soluble ST2 (sST2) attenuated IL-33 signaling, reduced regulatory T cells (Tregs) and ILC2s in adipose tissue, and promoted insulin resistance. Adipose-specific deletion of zinc finger and BTB domain–containing protein 7B further increased sST2 expression via an NF-κB–dependent mechanism, leading to inflammation, fibrosis, and impaired glucose homeostasis [110]. Age-related defects in adipose ILC2s further reduce thermogenesis and metabolic resilience [111]. Diet critically influences ILC2-dependent inflammatory regulation: ω-6 polyunsaturated fatty acids (PUFAs) promote airway inflammation, particularly in obesity, whereas ω-3 PUFAs can under certain conditions exacerbate allergic airway inflammation [43].

In contrast to adipose tissue, the lung is characterized by dynamic inflammatory fluctuations and variable substrate availability. Lung-resident ILC2s must rapidly respond to allergens and epithelial damage signals, proliferate locally, and produce cytokines, making regulation via metabolic checkpoints particularly important [9]. Upon IL-33 stimulation, pulmonary ILC2s preferentially take up exogenous FAs via CD36 under the control of PPARγ. Unlike adipose ILC2s, these lipids are transiently stored in LDs and subsequently utilized for phospholipid synthesis to support proliferation and pathogenic responses. These processes are tightly linked to glucose availability and mTOR signaling [9,34]. A recent study further demonstrated that the HIF-1α/glycolysis axis in ILCs regulates allergic airway inflammation [36]. Thus, pulmonary ILC2s integrate glucose as an enabling signal with FAs as structural and energetic substrate, and dietary glucose restriction attenuates ILC2-driven airway inflammation [9,34]. Lung ILC2s also rely on antioxidant systems, importing cystine to generate glutathione via glutathione peroxidase 4 (GPX4) and thioredoxin reductase 1 (TXNRD1), thereby increasing resistance to ferroptosis and oxidative stress [112].

Metabolic regulation in other tissues is less well characterized, but emerging evidence indicates tissue-specific adaptations. In the intestine, ILC2s are exposed to fluctuating nutrients and microbiota-derived metabolites such as SCFAs and bile acids [113]. Under conditions such as malnutrition or helminth infection, they utilize exogenous FAs and FAO-driven OXPHOS to maintain barrier protection and type 2 immunity, reflecting high metabolic flexibility [32,58]. Thus, intestinal ILCs must exhibit a high degree of “fuel flexibility” [113]. Recent work suggests that dietary fiber may be a critical determinant of pathological ILC2 responses in the gut [114]. In addition, IL-25 in the small intestine has been shown to promote long-term adaptive or memory-like states, which may also be linked to metabolic regulation [115].

In the skin, ILC2s are closely linked to barrier lipid metabolism and neuronal signaling pathways. Disruption of lipid homeostasis has been shown to amplify inflammation in an ILC2-dependent manner, and type 2 programs are GATA-dependent and associated with lipid synthesis [116]. Accordingly, LXR and PPARγ maintained lipid homeostasis and protected against psoriasis in mice models, whereas disruption of type 2 signaling promoted inflammation and metabolic imbalance [117].

In the brain, ILC2s reside in barrier-associated niches such as the choroid plexus and meninges, where they contribute to tissue homeostasis despite their low abundance [118,119]. With aging, these cells can accumulate and improve neuronal function while protecting against neurodegenerative diseases. Brain-resident ILC2s switch between quiescence and proliferation and are activated by IL-33, although their metabolic regulation remains poorly understood [118,119,120].

**Figure 4:**
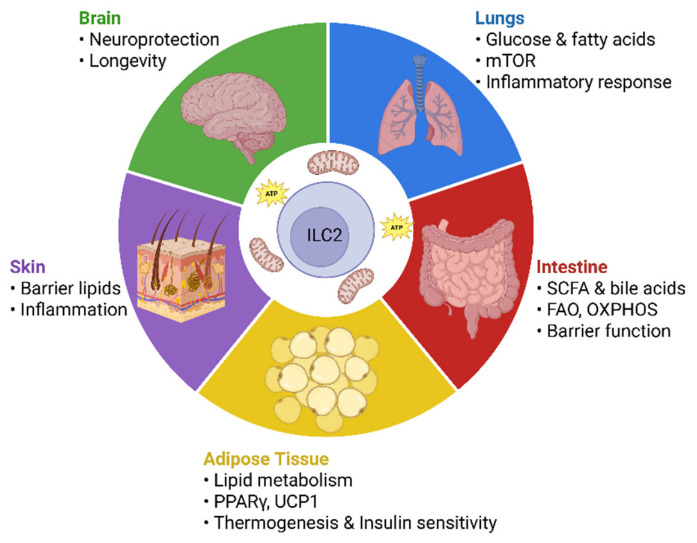
Tissue-Specific Metabolic Programs of ILC2s. Depending on the tissue microenvironment, ILC2s utilize distinct energy sources and signaling pathways that regulate their effector functions, inflammatory responses, and homeostatic roles. Created with BioRender.com (2026). Accessed on 10 April 2026.

Overall, ILC2s exhibit pronounced metabolic adaptability across tissues. Rather than relying on a single fuel source, they integrate available substrates, buffer lipid-induced stress, and couple metabolic pathways to effector functions in a context-dependent manner [113] (Figure 4).

## 8. Regulatory Nodes: Immunometabolic Checkpoints

**Figure 5:**
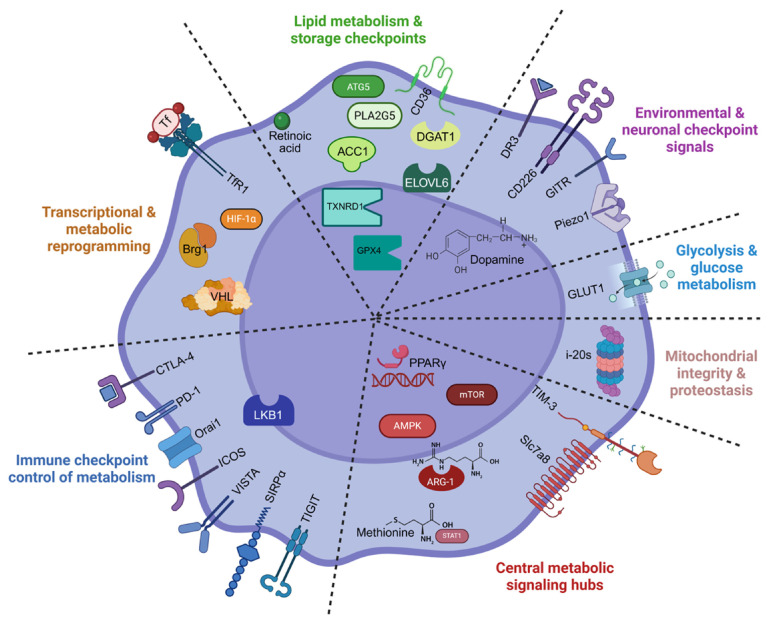
Key metabolic checkpoints in ILC2s. Immune checkpoints such as PD-1, CTLA-4, TIM-3, and ICOS modulate the balance between glycolysis, FAO, OXPHOS, and the TCA cycle, thereby shaping ILC2 proliferation, cytokine production, survival, and functional polarization. Created with BioRender.com (2026). Accessed on 2 May 2026.

Beyond core metabolic pathways, ILC2 metabolism is tightly controlled by immunometabolic checkpoints that act as functional “thermostats,” integrating nutrient availability, signaling pathways, and environmental cues to dynamically regulate glycolysis, FAO, the TCA cycle, and OXPHOS [11,48,53,55] (Figure 5, Table 2).

### 8.1. mTORC and AMPK as Master Regulatory Factors

The protein kinase mTOR forms two distinct complexes, mTORC1 and mTORC2, which orchestrate metabolic reprogramming in activated ILC2s. Its signaling promotes anabolic growth programs, including glycolysis, protein synthesis, and lipid biosynthesis, whereas AMPK senses energetic stress and shifts metabolism toward catabolic pathways such as FAO and mitochondrial maintenance [50,51]. mTOR regulates key processes such as protein, lipid, and nucleotide synthesis, mRNA translation, lysosomal biogenesis, and autophagy by phosphorylating targets including AKT, protein kinase C, and SREBPs. mTORC1 promotes anabolic metabolism in response to nutrients and inflammatory signals, driving glycolysis and biosynthesis while inhibiting autophagy [50,51,52]. In ILC2s, mTORC1 enhances PPARγ expression, supporting lipid metabolism, ST2 expression, and metabolic fitness, whereas mTORC2 contributes to glycolysis, cytoskeletal remodeling, proliferation, and survival [33]. In contrast, AMPK is activated under energy stress (high AMP/ADP, low ATP, ROS) and antagonizes mTOR signaling. AMPK promotes autophagy and a catabolic program characterized by increased OXPHOS and FAO, reduced lipid synthesis, and anti-inflammatory phenotypes [50,51]. Together, mTOR and AMPK function as a metabolic rheostat that balances anabolic glycolysis with catabolic FAO, aligning cellular growth and effector function with nutrient availability [47,51]. In this way, mTOR ensures that anabolic metabolism proceeds only when sufficient resources are available. The complex senses both nutrient availability and cellular energy status, balances the metabolic “tug-of-war” between anabolic glycolysis and catabolic FAO, and thereby ensures that cell growth, survival, and function are adapted to the environment. Dysregulation of this axis contributes to disease: hyperactive mTORC1 supports sustained glycolysis and proliferation, as observed in cancer and chronic inflammation, whereas insufficient mTOR activity impairs metabolic adaptation in aging and metabolic disorders [50,51,52]. In adaptive immunity, mTOR promotes differentiation of naïve CD4^+^ T cells into effector subsets, while its deficiency favors regulatory phenotypes [51]. In ILC2s, IL-33 activates the PI3K–AKT–mTOR pathway, enhancing nutrient uptake, glycolysis, lipid metabolism, and mitochondrial function. This leads to increased expression of glycolytic enzymes and glucose transporters, while FAO is simultaneously upregulated to support long-term energy supply and survival [51,121,122]. These pathways also provide biosynthetic intermediates required for proliferation and effector function [50,51]. Clinically, the IL-33/PI3K–AKT–mTORC1 axis has been linked to steroid-resistant asthma, potentially via upregulation of anti-apoptotic molecules such as B-cell lymphoma-extra large (Bcl-xL) and impaired GR signaling [123]. Conversely, inhibition of this pathway induces autophagy, reduces ILC2 accumulation, and attenuates tissue remodeling in eosinophilic chronic rhinosinusitis with nasal polyps (ECRSwNP) models [54,124].

### 8.2. Other Metabolic Checkpoints Regulating Lipid Metabolism in ILC2s

Metabolic checkpoints integrate nutrient availability, signaling pathways, and cellular energy status to fine-tune ILC2 function. Rather than acting in isolation, these regulators form an interconnected network that coordinates lipid metabolism, mitochondrial function, and effector responses.

Lipid biosynthesis pathways have been shown to influence ILC2 metabolic fitness. In murine models, acetyl-CoA carboxylase-1 (ACC1) was downregulated in ILC2s exposed to FAs from a high-fat diet, and pharmacological ACC1 inhibition impaired mitochondrial function while promoting adipose inflammation and accumulation of immature ILC2s [125]. Similarly, elongation of very long-chain FAs protein-6 (ELOVL6)-deficient mice exhibited increased sphingosine-1-phosphate levels and exacerbated airway inflammation, which could be partially reversed by sphingosine kinase inhibition [46].

Lipid storage and turnover have also been implicated as critical regulatory processes. In one study, loss of autophagy-related protein (ATG) 5 shifted metabolism toward glycolysis at the expense of FAO and TCA cycle activity, resulting in impaired cytokine production, reduced ST2 expression, and increased mitochondrial damage [126].

In addition, GPX4 and TXNRD1 were shown to protect ILC2s from lipid peroxidation, and GPX4 inhibition reduced ILC2 accumulation during allergic airway inflammation, suggesting a role for ferroptosis resistance in sustaining ILC2 responses [112].

Further evidence indicates that lipid sensing pathways contribute to ILC2 activation. Phospholipase A_2_ group V (PLA2G5) was reported to regulate expression of FFAR1, and its activation by extracellular FAs promoted ILC2 expansion [40].

In addition, micronutrients such as retinoic acid enhanced FAO and thermogenic programs in adipose ILC2s, whereas vitamin A deficiency increased ILC2 effector responses during parasitic infection [31].

Transcriptional regulators have also been linked to metabolic reprogramming in ILC2s. HIF-1α was shown to promote glycolysis following IL-33 stimulation, while deletion of von Hippel–Lindau (VHL) further enhanced HIF-1α–dependent glycolysis but impaired maturation of tissue-resident ILC2s [10,36,127]. Similarly, Brahma-related gene-1 (Brg1) increased chromatin accessibility of glycolytic genes, thereby promoting allergic inflammation [128].

Iron metabolism has emerged as an additional regulatory layer. Increased expression of transferrin receptor-1 (TfR1) enhanced iron uptake and mitochondrial metabolism during pulmonary ILC2 activation, whereas iron deprivation shifted metabolism toward HIF-1α–dependent glycolysis and reduced airway hyperresponsiveness in murine models [129].

Additionally, immune checkpoint pathways have been identified as central regulators of ILC2 metabolism. T-cell immunoglobulin and mucin-domain-containing 3 (TIM-3) has been associated with dysfunctional or exhaustion-like ILC2 states under chronic inflammatory conditions, suggesting a role in limiting sustained effector responses and metabolic overactivation [130]. Likewise, CD200-CD200R signaling has been implicated as an anti-inflammatory regulatory axis in type 2 immunity and may contribute to restraining ILC2 activation within tissue microenvironments [131]. Besides that, programmed cell death protein 1 (PD-1) was shown to limit survival and effector function by promoting apoptosis, whereas (PD-1) deficiency induced glycolysis, the pentose phosphate pathway, and amino acid metabolism while reducing OXPHOS, thereby enhancing proliferation and airway inflammation [132,133]. PD-1 expression itself appeared to be metabolically regulated, as impaired liver kinase B1 (LKB1) activity promoted mitochondrial dysfunction and features of metabolic exhaustion [134].

Checkpoint pathways further shape mitochondrial metabolism and cytokine responses. In one study, the Signal regulatory protein-alpha (SIRPα)–CD47 axis suppressed ILC2 activation and mitochondrial metabolism via Janus kinase (JAK/STAT) and MAPK signaling, whereas SIRPα deficiency increased TCA cycle activity and OXPHOS [92]. Deletion of the Ca^2+^ channels Orai1 and Orai2 markedly reduced glycolysis, FAO, and OXPHOS, impairing ILC2 effector function [135], while V-domain Ig suppressor of T-cell activation (VISTA) attenuated NF-κB signaling and ILC2 activity, and its deficiency enhanced mitochondrial metabolism [136]. Ciancaglini et al. showed that expression of Cytotoxic T-lymphocyte–associated protein-4 (CTLA-4) in tumor-associated ILC2s helps preserve mitochondrial respiratory capacity during chronic inflammation [137].

Environmental and neuronal signals have also been implicated in metabolic checkpoint regulation. Dopaminergic signaling via the D1 receptor suppressed OXPHOS and limited pulmonary ILC2 responses [138], whereas mechanosensory signaling through Piezo1 reduced mitochondrial activity. In contrast, activation of Glucocorticoid-induced tumor necrosis factor receptor (GITR) promoted glucose metabolism and ILC2 proliferation via NF-κB signaling [139].

Further metabolic regulators include CD226, whose blockade enhanced glycolysis while reducing FAO and lipid uptake, and Death receptor-3 (DR3), which activated NF-κB signaling and improved systemic glucose metabolism. Chronic airway inflammation has also been associated with dysfunctional T-cell immunoreceptor with Ig and immunoreceptor tyrosine-based inhibition motifs domains (TIGIT)-expressing ILC2s undergoing activation-induced cell death [140,141,142].

Finally, mitochondrial integrity has been linked to protein quality control systems. The immunoproteasome i-20s was shown to preserve mitochondrial capacity in ILC2s, whereas its inhibition disrupted mitochondrial metabolism and prevented IL-33–induced airway inflammation in murine models [61].

**Table 2:** Immunometabolic checkpoints and their Impact in ILC2s.

Metabolic Regulator	Metabolic Pathway Affected	Functional Consequence in ILC2	Disease Outcome	Sources
Lipid metabolism and storage checkpoints				
ACC1	Maintenance of lipid biosynthesis and mitochondrial function	Support of ILC2 metabolic homeostasis	Maintenance of ILC2 numbers	[27,143]
ELOVL6	Regulation of sphingolipid metabolism	Control of lipid-mediated inflammatory signaling	Deficiency aggravates allergic airway inflammation	[46]
ATG5	Glycolysis ↓ TCA, FAO ↑	Maintenance of metabolic homeostasis through autophagy	Support of ILC2 survival during allergic airway inflammation	[126]
GPX4 and TXNRD1	Lipid metabolism ↑, ferroptosis resistance	Protection of pathogenic ILC2s from lipid-induced cell death	Increase in ILC2 numbers in allergic airway inflammation	[112]
PLA2G5	Regulation of FA-receptor signaling (GPR40)	Promotion of macrophage-mediated ILC2 activation	Expansion of pulmonary ILC2 populations	[40]
Retinoic acid	FAO, Beiging and thermogenic lipid metabolism ↑	Modulation of tissue-specific ILC2 programs	Regulation of ILC2 function in metabolic tissues and lung	[30,66]
DGAT1	TAG synthesis and LD formation ↑	Prevention of lipotoxicity, buffering of excess FA influx, support of proliferation and activation under lipid-rich conditions	Promotion of pathogenic ILC2 expansion during airway inflammation	[34]
CD36	Exogenous FA uptake ↑	Facilitation of lipid uptake required for FAO or membrane lipid synthesis	Contribution to pathogenic airway inflammation, link to altered adipose lipid handling	[13,30]
Glycolysis and glucose metabolism				
GLUT1	Glucose uptake and glycolysis ↑	Support of activation-associated glycolysis, cytokine production, and proliferative responses	Promotion of allergic airway inflammation	[32,59]
Transcriptional and metabolic reprogramming				
HIF-1α	Glycolysis ↑	Metabolic activation and proliferation of ILC2s	Exacerbation of allergic airway inflammation	[36]
VHL	Glycolysis ↓	Promotion of maturation and tissue maintenance of ILC2s	Maintenance of pool of mature lung-resident ILC2s	[127]
Brg1	Glycolysis ↑	Enhancement of effector and memory-like ILC2 programs	Exacerbation of asthma	[128]
TfR1	OXPHOS via iron metabolism ↑	Enhancement of ILC2 activation and cytokine production	Promotion of airway hyperresponsiveness (AHR)	[11,129]
Immune checkpoint control of metabolism				
TIM-3	Glycolysis, mitochondrial metabolism ↓	Promotion of dysfunctional or exhausted ILC2 phenotype, limitation of sustained effector responses	Attenuation of chronic type-2 airway inflammation	[130]
CD200	Metabolic activation, proliferation, cytokine production ↓	Restriction of ILC2 activation and metabolic fitness	Reduction in AHR and allergic inflammation	[131]
PD-1	Apoptosis, Glycolysis, PPP, Glutaminolysis, Methionine catabolism ↓ OXPHOS ↑	Restriction of proliferation and cytokine production of ILC2s	PD-1 activation reduces AHR; PD-1 blockade enhances ILC2-driven inflammation	[132,133]
LKB1	Glycolysis, OXPHOS regulation	Maintenance of mitochondrial fitness and prevents exhaustion	Limitation of chronically activated or dysfunctional ILC2 responses	[134]
SIRPα	OXPHOS, TCA ↓	Suppression of ILC2 effector function	Reduction in AHR	[92]
Orai1 and Orai2	FAO, OXPHOS, Glycolysis ↑	Calcium-dependent metabolic activation of ILC2s	Enhancement of ILC2 effector function	[9,31]
VISTA	TCA, FAO, OXPHOS ↓	Inhibitory checkpoint limiting ILC2 metabolic activity	Reduction in airway inflammation	[48,113]
CTLA-4	Glycolysis ↓ Maintaining mitochondrial capacity	Negative regulation of ILC2 activation	Limit of excessive immune activation in inflammatory or tumor environments	[48,53]
ICOS	Regulation of cholesterol biosynthesis	Support of regulatory IL-10-producing ILC2 phenotype	Reduction in AHR	[15]
TIGIT	ILC2 exhaustion program	Induction of activation-induced cell death of ILC2s	Reduction in chronic allergic inflammation	[142]
Central metabolic signaling hubs				
mTOR	Autophagy, catabolic metabolism, OXPHOS, FAO ↓ Lipid biosynthesis ↑	Activation and expansion of pro-inflammatory effector cells	Promotion of pathogenic ILC2 activation during allergic airway inflammation, enhancement of AHR and eosinophilic lung inflammation, contribution to steroid-resistant asthma	[50,52,123]
AMPK	Autophagy, catabolic metabolism, OXPHOS, FAO ↑, Lipidbiosynthesis ↓	Promotion of regulatory/anti-inflammatory programs	Restriction of excessive ILC2 activation, Reduction in allergic airway inflammation and AHR, Support of metabolic adaptation under nutrient stress, limit of chronic pathogenic ILC2 responses	[38,144]
STAT3– Methionine axis	ATP-Synthesis, Methionin catabolism ↑	Support of mitochondrial energy production in ILC2s	Aggravation of allergic pneumonia	[60]
Slc7a8	Amino-acid transport sustaining mTOR and c-Myc signaling	Maintenance of metabolic fitness of ILC2s	Support of sustained ILC2 function	[47]
Arginase 1	Arginine metabolism, glycolysis↓	Metabolic checkpoint controlling ILC2 effector responses	Promotion of AHR	[59]
TIM-3	Glycolysis and mitochondrial metabolism ↓	Promotion of dysfunctional or exhausted ILC2 phenotype	Attenuation of sustained type-2 inflammatory responses	[134]
PPARγ	FA uptake ↑, FAO ↑, lipid metabolism ↑, ST2 expression ↑	Support of lipid uptake, metabolic fitness, IL-33 responsiveness, and IL-5/IL-13 production	Promotion of allergic airway inflammation and tissue-specific ILC2 activation, support of pathogenic or pro-tumoral ILC2 programs depending on context	[30,49]
Environmental and neuronal checkpoint signals				
Dopamine	OXPHOS ↓	Suppression of ILC2 activation	Reduction in allergic lung inflammation	[8]
Piezo 1	Mitochondrial metabolism ↓	Mechanosensitive suppression of ILC2 activation	Protection against AHR	[31]
GITR	Glucose metabolism regulation	Promotion of ILC2 proliferation and survival	Support of type-2 immune responses	[9,31]
CD226	Glycolysis ↓ FAO ↑	Modulation of metabolic activation of ILC2s	Contribution to AHR	[113]
DR3	Glucose metabolism regulation	Activation of naïve ILC2s and enhancement of cytokine production	Amplification of type-2 immune responses	[9,31]
Mitochondrial integrity and proteostasis				
Immunoproteasome i-20S	Preservation of mitochondrial capacity	Maintenance of metabolic fitness of ILC2s	Promotion of allergen-induced airway inflammation	[61]

↓ downregulated; ↑ upregulated

## 9. Discussion and Future Directions

Taken together, these findings highlight that metabolism, particularly FA and cholesterol metabolism in ILC2s, as well as membrane lipids and lipid raft organization serve functions far beyond merely structural or supportive roles. Importantly, lipids fundamentally shape the biophysical and biochemical properties of cellular membranes, including membrane organization, receptor clustering, and signaling platform formation, which are critical determinants of signaling strength and specificity. In addition, lipids contribute to key cellular processes such as signal transduction, energy metabolism, storage, and resistance to oxidative stress, thereby directly influencing ILC2 fate and function. Instead, they critically determine cytokine signaling sensitivity, for example to IL-33, and govern the functional polarization of ILC2s between pro-inflammatory type 2 responses and anti-inflammatory IL-10 production, thereby constituting central immunometabolic checkpoints in ILC2 biology [11,15,52,53,145]. ILC2 function is controlled by a highly integrated metabolic network in which carbon and lipid metabolism are inseparably linked. Collectively, these pathways enable ILC2s to adapt to distinct tissue environments, nutrient availability, and immunological challenges [11,31,53]. However, despite increasing insights into individual pathways, a comprehensive and quantitative “fuel map” defining substrate utilization and metabolic dependencies of ILC2s across different tissues remains lacking [31,113]. This represents a major conceptual gap, particularly given the pronounced tissue-specific heterogeneity and niche adaptation of ILC2s [1,5]. One example of a high-burden ILC2-driven disease is asthma, which affects approximately 5–10% of the global population and is characterized by airway obstruction, shortness of breath, and mucus overproduction [2,122]. Asthma is frequently driven by type 2 immune responses involving Th2 cells, eosinophils, mast cells, IgE, and lipid mediators such as leukotrienes [9,42]. In this context, ILC2s represent a key early source of type 2 cytokines and are critically shaped by metabolic inputs, including lipid availability and signaling pathways [2,11]. Current therapeutic strategies include bronchodilators such as α-sympathomimetics, as well as anti-inflammatory treatments including inhaled glucocorticoids and leukotriene receptor antagonists. In advanced disease stages, biologics such as IL-4Rα–blocking antibodies (e.g., dupilumab) are used [2]. However, approximately 30% of patients fail to achieve sustained clinical improvement, highlighting limitations of current therapies that primarily target downstream inflammatory pathways rather than upstream metabolic regulators [2,123,128]. These observations underscore the importance of immunometabolism as a therapeutic entry point. However, the translational maturity of proposed targets differs substantially. Some pathways, including mTOR signaling, PD-1, the HIF-1α/glycolysis axis, calcium-dependent metabolic activation, TIM-3, and CD200-CD200R signaling, already show robust preclinical evidence in murine models of allergic inflammation and airway hyperresponsiveness [36,52,123,130,131,132,133,134,135]. In contrast, other candidates such as cholesterol biosynthesis, lipid raft organization, ferroptosis resistance, or neuronal metabolic checkpoints remain at an earlier exploratory stage and require further mechanistic and in vivo validation [15,35,112,138]. Thus, future therapeutic development will likely depend on prioritizing context-specific targets with sufficient efficacy while minimizing systemic metabolic toxicity.

Metabolic pathways in ILC2s not only support cellular activation but actively determine the magnitude and persistence of type 2 inflammation, suggesting that targeting metabolic checkpoints could modulate disease at its root rather than merely alleviating symptoms [10,48]. At the same time, several challenges must be considered. Metabolic pathways are highly conserved and systemically interconnected, raising the risk that therapeutic targeting may disrupt global metabolic homeostasis or affect non-target cell populations [10,27]. Furthermore, inter-individual variability in metabolism driven by factors such as sex, age, diet, lifestyle, and physical fitness introduces an additional layer of complexity that may influence therapeutic responses and disease outcomes [10,144]. In addition, several major conceptual and technical barriers currently limit progress in the field. First, most metabolic insights are derived from bulk or in vitro systems, whereas ILC2s exhibit profound heterogeneity at the single-cell level, necessitating high-resolution approaches such as single-cell multi-omics to accurately define metabolic states across subsets and activation stages [12,113]. Second, strong tissue specificity of ILC2s poses a major challenge, as metabolic programs are highly context-dependent and shaped by local nutrient availability, stromal interactions, and organ-specific signals, limiting the generalizability of findings across tissues [1,5,31]. Third, substantial differences between murine and human ILC2 biology complicate translational efforts, particularly with respect to metabolic wiring and responsiveness to environmental cues [12,48]. Finally, a critical unresolved issue is how to achieve therapeutic specificity when targeting metabolic pathways, given their ubiquitous role across cell types; strategies will need to exploit context-dependent vulnerabilities, cell-type–restricted regulators, or spatial targeting to avoid systemic toxicity and preserve physiological homeostasis [10,27,48]. Ultimately, future research should aim to define tissue-specific metabolic programs, identify context-dependent vulnerabilities, and develop strategies that allow selective modulation of ILC2 metabolism without compromising systemic homeostasis. Mapping these pathways in a tissue- and disease-specific manner will be essential to establish a functional “metabolic atlas” of ILC2s and to guide the rational design of targeted therapies [31,113].

## 10. Conclusions

ILC2s exhibit dynamic metabolic plasticity, integrating glycolysis, OXPHOS, and FAO. Metabolic intermediates play a critical role in immune function and essential cellular processes, rather than serving solely as sources of energy. Targeted modulation of immunometabolism may therefore represent a transformative therapeutic strategy for airway diseases, metabolic disorders, and cancer.

## Figures and Tables

**Table 1:** Lipids in ILC2s.

Lipid Class	Sites of Production in ILC2s	Functions in ILC2s	Source
Free FAs	dietary lipids adipose tissue lipolysis synthesis in cytosol uptake into ILC2s from extracellular milieu	major fuel source for FAO, ATP generation, and mitochondrial fitness; support of ILC2 activation and proliferation under nutrient-adaptive conditions.	[11,13,32]
Triacylglycerols (TAGs)	intracellularly in activated ILC2s from imported FAs	transient lipid storage depot that buffers FA availability and supplies substrates for FAO and membrane biosynthesis during ILC2 expansion and effector responses	[34,37]
Phospholipids	sER and cellular membranes generation partly from stored and imported FAs	structural membrane components required for biomass accumulation, membrane remodeling, organelle expansion, and signaling platform formation during ILC2 activation	[35,38]
Cholesterol/membrane sterols	de novo synthesis in cytosol and sER uptake from extracellular lipoproteins	stabilization of the membrane organization and lipid rafts, thereby facilitating receptor clustering and downstream signaling relevant to ILC2 activation; serves as a precursor for numerous bioactive molecules, including oxysterols, steroid hormones (e.g., cortisol), bile acids, and vitamin D and A metabolites; transcriptional regulation of cholesterol homeostasis is mediated by SREBPs and LXRs, which control the expression of enzymes such as HMGCR, receptors such as LDLR, and ABC transporters	[15,26,39]
Eicosanoids (general)	produced locally from arachidonic acid mainly by mast cells, eosinophils, macrophages, epithelial cells, and other inflamed tissue cells	fine-tuning of ILC2 activation, migration, cytokine secretion, and tissue inflammation	[9,40]
PGD2	primarily mast cells other myeloid cells in inflamed tissue	activation of ILC2s: promotion of chemotaxis and enhancement of type 2 cytokine production	[41]
Cysteinyl leukotrienes (LTC4, LTD4, LTE4)	primarily mast cells eosinophils, basophils, and other leukocytes	activation of ILC2s: enhancement of cytokine production, proliferation, and airway inflammatory responses	[42]
LTB4	myeloid cells, especially neutrophils, macrophages, mast cells	support of ILC2 recruitment and inflammatory activation	[9]
PGI2	endothelial cells and other stromal/tissue cells	negative regulator of ILC2: suppression of cytokine production and dampening of allergic airway inflammation	[9]
Lipoxin A4	generated during resolution phase by leukocyte–leukocyte or leukocyte–epithelial transcellular pathways	pro-resolving lipid mediator that inhibits ILC2 activation and limits type 2 inflammation	[9]
Maresin-1/pro-resolving omega-3 lipid mediators	derived from omega-3 FAs, mainly by macrophages during inflammatory resolution	suppression of ILC2-driven inflammation and promotion of return to tissue homeostasis	[9,43]
Sphingolipids/ceramides	De novo synthesis in ER/Golgi and membrane turnover in many host cells	contribution to membrane organization, signal transduction, and inflammatory set points	[44,45,46]

## Data Availability

No new data were created or analyzed in this study. Data sharing is not applicable to this article.

## Abbreviations

ACC1	acetyl-CoA carboxylase 1
AhR	aryl hydrocarbon receptor
AHR	airway hyperresponsiveness
AKT	protein kinase B
AMPK	adenosine monophosphate -activated protein kinase
AP-1	activator protein 1
ARG1	arginase1
ATG5	autophagy-related protein 5
ATP	adenosine triphosphate
BCAAs	branched-chain amino acids
Bcl-xL	B-cell lymphoma-extra large
Blimp-1	B-lymphocyte-induced maturation protein 1
cAMP	cyclic adenosine monophosphate
c-MAF	MAF bZIP transcription factor
COX	cyclooxygenase
CPT1	carnitine palmitoyltransferase 1
CTLA-4	cytotoxic T-lymphocyte-associated protein 4
DGAT1	diacylglycerol O-acyltransferase 1
DR3	death receptor 3
ECRSwNP	eosinophilic chronic rhinosinusitis with nasal polyps
ELOVL6	elongation of very long-chain fatty acids protein 6
ER/sER	endoplasmic reticulum/smooth endoplasmic reticulum
FAO	fatty acid oxidation
FA/FAs	Fatty acid/fatty acids
FATPs	fatty acid transport proteins
FFAR 1/FFAR 2/FFAR 3	free fatty acid receptor 1/2/3
GITR	glucocorticoid-induced tumor necrosis factor receptor
GLUT1	glucose transporter 1
GPI	glycosylphosphatidylinositol
GPX4	glutathione peroxidase 4
GR	glucocorticoid receptor
HDACs	histone deacetylases
HIF-1α	hypoxia-inducible factor 1 alpha
ICOS	inducible T-cell costimulator
IgA	immunoglobulin A
ILCs/ILC2s	innate lymphoid cells/group 2 innate lymphoid cells
IL-1RAcP	interleukin-1 receptor accessory protein
IL	interleukin
JAK	Janus kinase
JNK	c-Jun N-terminal kinase
LDs	lipid droplets
LKB1	liver kinase B1
LXR	liver X receptor
MAPK	mitogen-activated protein kinase
MEF2C	myocyte enhancer factor 2C
mTOR	mechanistic target of rapamycin
mTORC1/2	mechanistic target of rapamycin complex 1/2
NADH	nicotinamide adenine dinucleotide
NADPH	nicotinamide adenine dinucleotide phosphate
NF-κB	nuclear factor kappa-light-chain-enhancer of activated B cells
NFAT	nuclear factor of activated T cells
NFIL3	nuclear factor interleukin-3-regulated
OXPHOS	oxidative phosphorylation
PD-1	programmed cell death protein 1
PD-L1	programmed death-ligand 1
PDK1	phosphoinositide-dependent kinase 1
PGs/PGE2/PGD2/PGI2	prostaglandins/E2/D2/I2
PI3K	phosphoinositide 3-kinase
PIP_2_	phosphatidylinositol 4,5-bisphosphate
PIP_3_	phosphatidylinositol 3,4,5-trisphosphate
PKA	protein kinase A
PLA2G5	phospholipase A2 group V
PPARγ	peroxisome proliferator-activated receptor gamma
PPP	pentose phosphate pathway
PUFAs	polyunsaturated fatty acids
ROS	reactive oxygen species
SCFAs	short-chain fatty acids
SIRPα	signal regulatory protein alpha
S1P	sphingosine-1-phosphate
S1PR1–5	sphingosine-1-phosphate receptors 1–5
SREBPs	sterol regulatory element-binding proteins
sST2	soluble ST2
STAT 3/STAT5	signal transducer and activator of transcription 3/5
TAGs	triacylglycerols
TCA	tricarboxylic acid
TfR1	transferrin receptor1
TIGIT	T-cell immunoreceptor with Ig and immunoreceptor tyrosine-based inhibition motif domains
TIM-3	T-cell immunoglobulin and mucin-domain-containing 3
TSLP	thymic stromal lymphopoietin
TXNRD1	thioredoxin reductase 1
UCP 1/UCP 2	uncoupling protein 1/2
VHL	von Hippel–Lindau
VISTA	V-domain Ig suppressor of T-cell activation
WAT	white adipose tissue
